# Exploring the Effect of Compound Glycyrrhizin and Silybinin on the Metabolism of Pexidartinib in Rats Based on CYP3A4 and CYP2C9

**DOI:** 10.1155/2023/6737062

**Published:** 2023-12-01

**Authors:** Yanding Su, Xinyi Wei, Qian Cheng, He Qi, Jianghui Chen, Xiang-Jun Qiu

**Affiliations:** College of Basic Medicine and Forensic Medicine, Henan University of Science and Technology, Luoyang 471023, China

## Abstract

Pexidartinib offered a new therapeutic option for adult patients with symptomatic tenosynovial giant cell tumor (TGCT) who were refractory to surgical treatment and had severe morbidity or functional limitations. Meanwhile, the metabolism of pexidartinib was mainly mediated through the oxidation of cytochrome P450 (CYP) 3A and glucuronidation by uridine glucuronosyltransferase (UGT) 1A4 and attention shall be paid to CYP450-based drug-drug interactions during therapeutic dosing. This study aimed to examine the changes in the pharmacokinetics of pexidartinib by silymarin and compound glycyrrhizin on pexidartinib in vivo in rats by high-performance liquid chromatography (HPLC)-UV approach and to detect its expression in CYP3A4 and CYP2C9 using the western blot. The findings of chromatography experiments revealed that silybinin as well as compound glycyrrhizin increased the exposure of pexidartinib in rats and had a significant inhibitory effect on the metabolism of pexidartinib. The results of immunoblotting assays suggested that silybinin as well as compound glycyrrhizin inhibited the protein expression of CYP3A4 and CYP2C9 in rats. Therefore, the combination of pexidartinib with silybinin and compound glycyrrhizin should be monitored to avoid clinical adverse effects.

## 1. Introduction

With the rapid development of clinical careers, Western medicine combined with TCM treatment has become an increasingly common practice. In the treatment of clinical tumors, Western medical treatment is often accompanied by combination therapy with TCM, which can provide a reasonable treatment plan to improve the immunity of patients and play a role in reducing toxic side effects and improving their quality of life [1]. However, often drug-drug interactions (DDIs) with oral antineoplastic agents (OAs) cannot be avoided during clinical combination therapy, so clinicians should always pay attention when screening for DDIs in OAs [2].

Pexidartinib (PXD) as an oral small molecule tyrosine kinase inhibitor (TKI) [3] will provide a new therapeutic option for adult patients with symptomatic TGCT who are refractory to surgical treatment and have severe morbidity or functional limitations [4]. PXD selectively inhibits CSF-1R, c-KIT proto-oncogene receptor tyrosine kinase (KIT), and Fms-like tyrosine kinase 3 internal tandem duplication mutation sinFms-like tyrosine kinase-3 (FLT3-ITD), thus acting as an inhibitor of tumor cell proliferation [5]. Although the use of PXD avoids the significant morbidity associated with conventional treatment regimens, there are clear risks associated with the treatment of PXD. Due to the potentially lethal nature of the severe liver injury associated with PXD [6], timely monitoring of liver function is often required for dose adjustment at the time of administration.

Silibinin is derived from Silybummarianum, a plant of the Asteraceae family, and is a flavonolignan compound with significant antioxidant, anti-inflammatory properties, and hepatoprotective and anticancer activities [7, 8]. Silibinin, as the main active component of silymarin [9], has good efficacy against drug-related liver injury [10] and is widely used in drug-related liver injury [11]. Compound glycyrrhizin (CG) is a glycyrrhizin tablet made from glycyrrhizin, the active ingredient in licorice, with DL-methionine and glycine [12], which is widely used in the clinic for drug-induced liver injury [13, 14]. Glycyrrhizin possesses the characteristics of protecting cell membranes, reducing aminotransferases, improving liver function, and repairing liver tissue damage [15]. Glycyrrhizin can be decomposed into pharmacologically active glycyrrhizinic acid in vivo to exert anti-inflammatory, immunomodulatory, and hepatocyte membrane protection effects [16]. Studies have shown that silibinin [17] and glycyrrhizinic acid [18] can inhibit the activities of CYP3A4 and CYP2C9 enzymes. PXD metabolism is mainly mediated through the oxidation of cytochrome P450 (CYP) 3A and the glucuronidation of uridine glucuronosyl transferase (UGT) 1A4 [19], so it is important to pay attention to the effects of silibinin and CG when combining with PXD, respectively. Attention should be paid to the CYP450-based DDI of both.

Therefore, this research developed and demonstrated an accurately and rapidly performed HPLC-UV approach for the determination of PXD in rat plasma with a facile liquid-liquid extraction protocol to evaluate the changes of silybinin and CG on the pharmacokinetics of PXD in rats. Meanwhile, we used western blot to measure the expression of CYP3A4 and CYP2C9 to investigate the mechanism of the effect of the two on the metabolism of PXD in rats and to provide a theoretical guide for the clinic.

## 2. Materials and Methods

### 2.1. Chemical Materials

**Figure 1:**
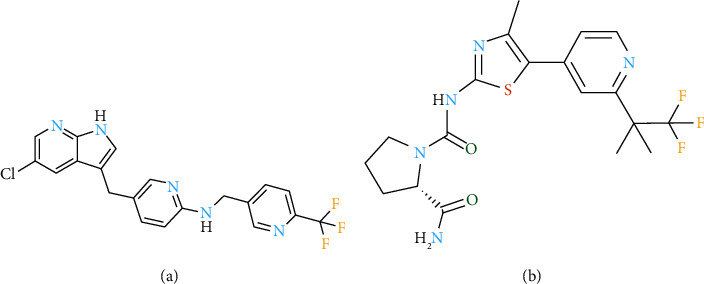
Chemical constitution of PXD (a) and alpelisib (b).

PXD (Figure 1 compound A, purity >98%), CAS1029044-16-3, and alpelisib (Figure 1 compound B, internal standard, purity >98%), CAS1217486-61-7. They were all obtained by Shanghai Lou Lan Biological Technology Co. Ltd. Silybinin capsule (State Pharmaceutical License H20040299) was purchased from Tianshili Pharmaceutical Group Co. Ltd, compound glycyrrhizin tablet (State Pharmaceutical License H20083001) was purchased from Beijing Kain Science and Technology Co. Ltd, PMSF Solution (ST507-10 ml), and RIPA lysate (P00138), PVDF membrane (FFP24), BCA protein concentration assay kit (P0012) were purchased from Shanghai Biyuntian Biotechnology Co. Horseradish enzyme-labeled goat anti-rabbit IgG (H + L) was purchased from Beijing Zhongsui Jinqiao Bio-technology Co. Actin monoclonal antibody was purchased from Wuhan Sanying Biotechnology Co. The Efficient Chemiluminescence kit (GE2301-100 ml) was purchased from gen-view Technology Co., and the antibodies to CYP3A4 (ab124921) and CYP2C9 (ab150364) were obtained from Abcam. Chromatographically pure methanol and acetonitrile were available from the Tianjin Komeo Chemical Reagent Development Center. Ultrapure water was produced via filtration through a Milli-Q reagent system (Bedford Millipore, USA).

### 2.2. Animal

Thirty-three clean-grade male SD rats, with a body mass of 200 ± 20 g, were offered by the Experimental Animal Center, School of Basic Medicine and Forensic Sciences, Henan University of Science and Technology. Animal license number: SCXK(E) 2019-0002. Experiments were performed after a 7-days acclimatization feeding, and the animals were fasted and dehydrated for 24 h before the experiment. Animal experiments were conducted following the Principles of Animal Experimentation (National Science and Technology Commission of China).

In the chromatography experiment, 18 rats were randomly divided into the CG group (group C, 6 rats), silybinin group (group B, 6 rats), and control group (group A, 6 rats), and all three groups were gavaged once a day for fourteen consecutive days. Group B was gavaged with silybinin aqueous solution 150 mg/kg (containing silybinin 21 mg/kg), group C was gavaged with compound glycyrrhizin aqueous solution 13.5 mg/kg (containing glycyrrhizin 4.5 mg/kg), and in contrast, group A was gavaged with an equal amount of saline. After 14-days of administration, three groups were given 40 mg/kg PXD. 0.5 mL of blood was taken from the caudal vein at 0.25, 0.50, 1.00, 2.00, 3.00, 4.00, 6.00, 8.00, 12.00, 24.00, and 48.00 h after being gavaged and added to EP tubes containing heparin, followed by centrifugation at 3500 rpm for 15 min, and finally the supernatant was stored at −20°C and stored for measurement.

In the western blot experiment, 15 rats were randomly divided into a control group (group D, 3 rats), a silybinin group (group E, 3 rats), CG group (group F, 3 rats), a silybinin + PXD group (group G, 3 rats), and a CG + PXD group (group H, 3 rats). The rats in all five groups were gavaged once a day for fourteen consecutive days. Groups E and G were gavaged with 150 mg/kg aqueous solution of silybinin (containing 21 mg/kg silybinin), groups F and H were gavaged with 13.5 mg/kg aqueous solution of compound glycyrrhizin (containing 4.5 mg/kg glycyrrhizin), and from the 15-days onwards, groups G and H were given 40 mg/kg of PXD per day for a total of 7-days, and equal amounts of saline were given to groups E and F. Group D was gavaged with equal amounts of saline until the end of the experiment. After gavage for 21 days, rats in each group were anesthetized with 10% urethane, and their fresh livers were snap-frozen in liquid nitrogen and stored at −80°C for subsequent experiments.

### 2.3. Chromatographic Instrumentation and Analysis

The apparatus used in the chromatographic analysis of this work was an 1100 series high-performance liquid chromatography (product of Agilent, USA), which mainly included a vacuum degasser, a four-component infusion pump, an automatic sampler, a column temperature box, and a DAD detector. The fundamental steps were as follows. First of all, the chromatographic separation was carried out on a Venusil C18 column (4.6 mm × 250 mm, 5 *μ*m). Subsequently, the analytes were thoroughly separated under the mobile phase water, acetonitrile, and 0.2% TFA (37 : 43 : 20).

### 2.4. Preparation of Standard Products

Configure the corresponding 1 mg/ml standard stock solution and internal standard stock solution by adding 5 ml of methanol to 5 mg of PXD and 5 mg of alpelisib samples, respectively, after which both were progressively diluted with methanol into 100 ng/ml, 10 ng/ml, and 1 ng/ml working solutions. The above were refrigerated and kept at 4°C.

The manipulation of plasma standards was accomplished with the configured PXD working solution: 100 *μ*l of the correspondent working solution and 900 *μ*l of rat blank plasma were filled in the EP tubes. After that, PXD calibration standards with the following concentrations were sequentially made: 0.05, 0.1, 0.25, 0.5, 1, 2.5, 5, and 10 *μ*g/ml. In the end, three quality control (QC) samples of low, medium, and, high (0.1, 2.5, 7.5 *μ*g/ml) were constructed in various concentrations of working solution and blank plasma.

### 2.5. Sample Handling

After preparatory 2 ml EP tubes, 300 *μ*L of plasma samples and 15 *μ*L of 10 ng/ml of alpelisib working solution were pipetted separately, followed by 300 *μ*L of 10% sodium carbonate solution, mixed well, and then 1.0 ml of ethyl acetate was included to sediment the proteins. The mixture was vortexed for 2.0 min and subsequently centrifuged at 3500 rpm for 20 min at 4°C. At last, 900 *μ*L of the upper organic phase was transferred to a fresh EP tube, blown dry with an air stream at 50°C, and then redissolved with 100 *μ*L of mobile phase, and 20 *μ*L was sampled for the assay.

### 2.6. Methodological Examination

Based on the “Guidelines for the Validation of Quantitative Methods for the Analysis of Biological Samples” in the Chinese Pharmacopoeia 2020, Part IV General Provisions, the selectivity, standard curve, matrix effect and stability, accuracy, and precision of the proposed method were validated.

The empty plasma supplemented with silybinin was manipulated as mentioned above for plasma specimen treatment and then injected into 20 *μ*L for assay. The blank plasma and the plasma supplemented with PXD and ISTD were prepared in the same way to get the corresponding chromatograms. The specificity of the approach was assessed by the comparison of the chromatograms of the three to ensure the absence of endogenous interference.

PXD plasma standard solutions with 0.05, 0.1, 0.25, 0.5, 1, 2.5, 5, and 10 *μ*g/ml were configured, treated according to the above plasma sample handling procedure and then assayed on the machine for testing, Finally, the regression equation of PXD was calculated by linear regression of PXD concentration as *X*-axis, with the ratio of PXD peak area (As) to ISTD peak area (Ai) as *Y*-axis.

PXD QC samples were formulated at levels of 0.1, 2.5, and 7.5 *μ*g/ml, and 6 samples of each concentration were used in parallel and assayed according to the “plasma sample processing method.” The intraday precision was calculated on the following day, and the interday precision was calculated in 3 sequential days.

The PXD QC samples were configured separately at concentrations of 0.1, 2.5, and 7.5 *μ*g/ml, respectively, and six copies of each were allocated in parallel. The extraction recoveries were calculated as the ratio of the peak area of PXD in plasma to the peak area in the corresponding mass concentration control solution.

The stability of PXD was investigated under the following four storage conditions via the repeated prep of six parallel samples each of 0.1, 2.5, and 7.5 *μ*g/ml of PXD QC: (1) 4 h at room temperature. (2) 24 h at 4°C. (3) Three cycles of freeze-thawing of the samples. (4) 2 weeks at −20°C.

### 2.7. Immunoblot Analysis

Fresh livers were removed at −80°C and weighed portions were homogenized in ep tubes with lysis buffer containing 0.1% PMSF. We prepared 12% SDS-PAGE routine gel, then each group took the corresponding protein samples and prestained Marker samples, 80 V constant voltage for 90 min, the voltage was adjusted to 120 V, and electrophoresis was continued for 30 min. After electrophoresis, the membrane transfer “Sandwich” was constructed in the order of filter paper, PVDF membrane, gel and filter paper from bottom to top, and the membrane transfer was carried out after the construction was completed. After the end of membrane transfer, each group was closed at room temperature for 2 h with 5% skim milk powder, washed five times with TBST and added with the corresponding primary antibodies CYP3A4 (1 : 5000) and CYP2C9 (1 : 5000), and incubated at 4°C overnight. After removing and washing five times with TBST on the next day, the corresponding secondary antibody was added and incubated for 1 hour at room temperature. The membranes were then washed with TBST, and given 200 *μ*l of Efficient Chemiluminescence solution before going on the machine for detection. Imaging was performed using Imgage-ProPlus 6.0 software and quantitative analysis was performed using Image J software. The results were expressed as the ratio of the relative intensity of the target protein to the relative intensity of the internal standard protein.

### 2.8. Plasma Sample Testing and Data Handling Analysis

Plasma specimens were assayed in a batch process consisting of two analytical batches, each accompanied by a standard curve and quality control specimens. When there exceeded the upper line concentration point, the test would need to be diluted with a blank matrix and multiplied by the dilution factor to obtain the final concentration.

In the current study, the drug concentration of PXD in rat plasma was plotted versus the time curve as well as all the bar graphs using GraphPad Prism 8.0 software. Then, DAS (Drug And Statistics) 2.0 software (Shanghai University of Traditional Chinese Medicine, China) was employed to get the main pharmacokinetic parameters of the analytes in nonatrial mode. Then, a one-way analysis of variance (ANOVA) was performed using SPSS (statistical product and service solutions) 25.0 software, while finally, an independent samples *t*-test was applied to the comparison of major pharmacokinetic parameters between the two groups, where Tmax was tested using rank sum test. Statistical significance was indicated by *P*  <  0.05.

## 3. Findings and Discussion

### 3.1. Specificity

**Figure 2:**
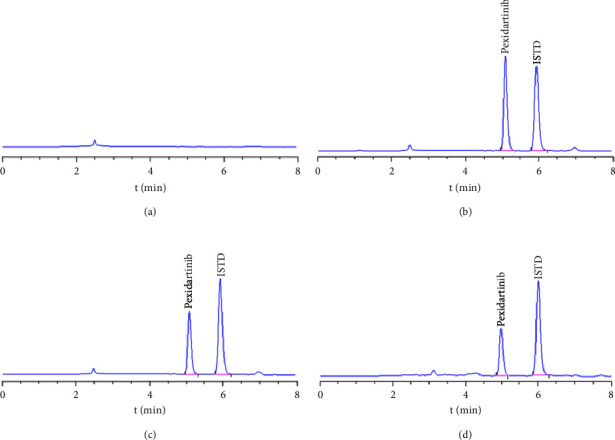
Representative chromatograms of PXD and ISTD: (a). The blank plasma; (b). The blank plasma adulterated with PXD and ISTD; (c). The specimens were taken from rats after oral dosing of 40 mg/kg PXD for 4.0 h in group B; (d). The specimens were taken from rats after oral dosing of 40 mg/kg PXD for 4.0 h in group C.

Under the chromatographic conditions of this experiment, the retention time of PXD was about 5.094 min and that of ISTD was about 5.953 min. The three were well separated, no spurious peaks interfered with the determination, and the baseline was smooth, as illustrated in Figure 2. The current approach had high specificity and could accurately determine the concentration of PXD in plasma with good reproducibility.

### 3.2. Standard Curve and Lower Limit of Quantification

**Figure 3:**
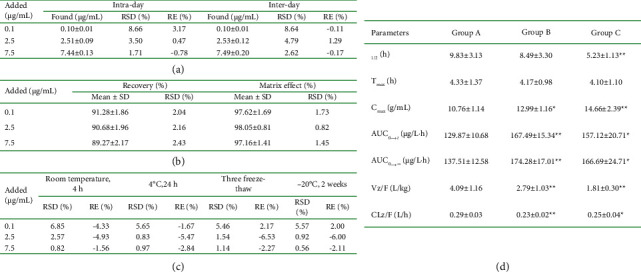
(a): The accuracy, and precision of PXD in rat plasma (*n* = 6); (b): recovery and matrix effect of PXD from rat plasma (*n* = 6); (c): stability of PXD in rat plasma subjected to various conditions (*n* = 6); (d): major pharmacokinetic properties of PXD in rats (*n* = 6, mean ± SD).

**Figure 4:**
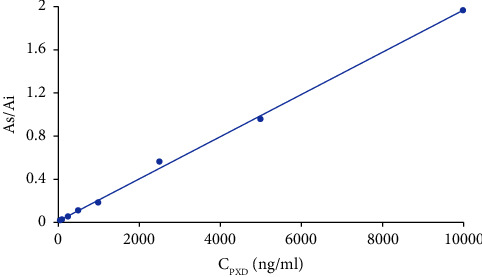
Calibration curve of PXD.

As indicated in Figure 3(a), during this experiment, eight different concentrations of the calibration curve from 0.05–10 *μ*g/mL were incorporated into a blank sample of rat plasma and then evaluated by HPLC-UV for three consecutive days. The standard curve (Figure 4) for PXD was well linear in the range of 0.05–10 *μ*g/mL with a regression equation of *Y* = 1.96 × 10^−4^ × X + 3.99 × 10^−3^ (*r*^2^ = 0.9991). Finally, the lower limit of quantification (LLOQ), as the lowest concentration of the analyte in the specimen that was able to be measured reliably, had a value of 0.05 *μ*g/mL in the present research, with RSD 6.72% and RE -3.67%.

### 3.3. Accuracy and Precision

As presented in Figure 3(a), the accuracy and precision of PXD were calculated by performing several replicate assays and detailed analysis at 0.1, 2.5, and 7.5 *μ*g/ml. All of the obtained values were within ±15%, which indicates that the measurement of PXD concentration in rat plasma was done reliably and accurately with this approach.

### 3.4. Recovery and Matrix Effect

In Figure 3(b), in rat plasma, it could be derived that the average extraction recoveries of analytes in QC specimens of 0.1, 2.5, and 7.5 *μ*g/ml were 87.10%–93.14%, indicating the high reproducibility of the proposed approach.

### 3.5. Stability

The stability of the test samples was examined and characterized at 0.1, 2.5, and 7.5 *μ*g/ml (Figures 3, 3(c)), which demonstrated that the measurements were stable at room temperature (short-term), 4°C (long-term), repeated freeze-thaw, and −20°C (2 weeks). The RSD was less than 10% for all conditions of storage, which complied with the above-mentioned “Guidelines for the validation of quantitative methods for the analysis of biological samples”.

### 3.6. Pharmacokinetic Study

**Figure 5:**
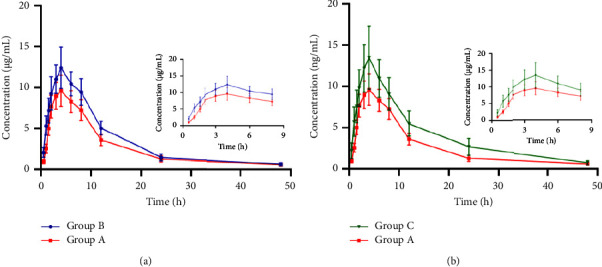
Mean plasma concentration-time curve of PXD in two groups of rats (*n* = 6).

A freshly established UPLC-UV bioassay was employed to examine the pharmacokinetics of PXD with silybinin and CG in rats. The average concentration values of PXD versus time in the plasma of rats in the silybinin group as well as in the CG group versus time after a single oral dose of 40 mg/kg PXD were plotted in Figure 5, and the primary pharmacokinetic parameters of PXD were described in Figure 3(d). Group B exhibited significantly elevated Cmax (*P*  <  0.05), AUC_0⟶t_, and AUC_0⟶∞_(*P*  <  0.01) compared to group A, with increases of 20.72%, 28.97%, and 26.74%, respectively. While *Vz*/*F*(*P*  <  0.01) and *CLz*/*F*(*P*  <  0.05) were dramatically decreased, with a decrease of 31.78% and 20.69%, respectively. *C*_max_(*P*  <  0.01), AUC_0⟶t_, and AUC_0⟶∞_(*P*  <  0.05) were markedly augmented in group C than in group A, which increased by 36.25%, 20.98%, and 21.22%, individually. Whereas, *t*1/2 (*P*  <  0.01), *Vz*/*F*(*P*  <  0.01), and *CLz*/*F*(*P*  <  0.05) were decreased significantly by 31.78% and 20.69%, correspondingly. This indicated that silybinin, CG could influence the pharmacokinetics of PXD in rats and enhance the plasma exposure of PXD.

### 3.7. Western Blot

**Figure 6:**
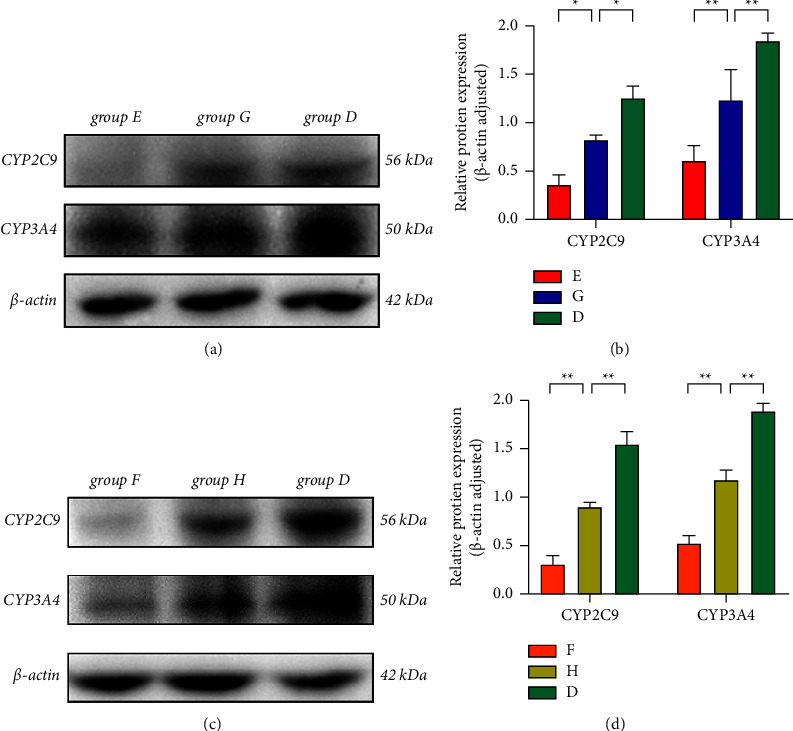
Protein expression of CYP3A4 (50 kDa) as well as CYP2C9 (56 kDa) (a, b) and the ratio of the gray value of the target band to the gray value of the *β*-actin band (c, d) for each group, ^*∗*^*P*  <  0.05 and ^*∗∗*^*P*  <  0.01.

Western blot findings (Figure 6) indicated that the protein expression levels of CYP3A4 and CYP2C9 were decreased in the silybinin group, silybinin + PXD, CG group, and CG + PXD group as compared with the control group (*P*  <  0.05 and *P*  <  0.01, respectively), which suggests that the effects of silybinin and PXD in combination and CG and PXD in combination on the metabolism of PXD in rats are related to the inhibition of CYP3A4 and CYP2C9.

## 4. Discussion

### 4.1. Chromatographic Methodology Development and Optimization

The analytes were measured at a wavelength of 254 nm at the beginning of this research, and the results were acquired by checking the 3D pattern to search for the optimum absorption wavelength for this analysis, which revealed that the maximum peak value of the analytes was achieved at a wavelength of 306 nm. Finally, the ZORBAX XDB-C18 column (150 mm × 4.6 mm, 5.0 *μ*m) and Venusil C18 column (250 mm × 4.6 mm, 5.0 *μ*m) were used for comparison, showing that the latter had better separation and reproducibility under the same analytical conditions, leading to better reproducible analytical results.

Due to the high blood concentration of PXD, protein precipitation, perchloric acid extraction, and ethyl acetate extraction approaches were initially compared in this experimental, whose results revealed that the first two were not satisfactory, probably related to its high plasma protein binding rate [20]. Therefore, the ethyl acetate extraction process was finally used in this experiment.

Acetonitrile possessed the characteristics of both low column pressure and high mass spectrometric response value, so it was employed as the organic phase in this study. Then, the present experiment was conducted to compare the effects of water-acetonitrile, methanol-acetonitrile, and methanol-acetonitrile-0.2% TFA on the responses of the target compounds. As a result, it was found that the mobile phase of methanol-acetonitrile-0.2% TFA showed a high response of the target analytes with good specificity and no peak dragging.

### 4.2. DDIs

The most frequent adverse events associated with PXD, a small molecule TKI, were hair hypopigmentation or transient transaminase elevation, and serious or permanent adverse events, especially cholestatic hepatotoxicity, were rare [21]. DDIs in the PXD were reported when combined with the sedative-hypnotic drug midazolam, the cardiac glycoside analog digoxin, the proton pump inhibitor omeprazole [19], and the antifungal drugs fluconazole and itraconazole [22]. The PXD carried a risk of fatal hepatic injury, so prompt hepatoprotective therapy was essential. Silybinin possessed both hepatoprotective effects and therapeutic pigmentation hypopigmentation [7], and as with CG, both represented excellent therapeutic agents for drug-induced liver injury. When combined with PXD for hepatoprotective therapy, both could cause DDIs.

Currently, there are no clinical reports on the detection approaches of PXD and silibinin as well as CG in biological samples. Therefore, in this research, we developed and demonstrated an accurately and rapidly performed HPLC-UV approach. In the pharmacokinetics findings, after a single oral dose of PXD, *C*_max_, AUC_0⟶t_, and AUC_0⟶∞_ were all markedly higher in the silybinin as well as CG groups, demonstrating that silybinin, as well as CG, had a more pronounced inhibitory effect on the metabolism of PXD, which may be related to the inhibition of the protein expression of CYP3A4 and CYP2C9 by silybinin as well as CG. Then, we verified the above hypothesis in western blot assay, and it showed that the two drugs did depress the protein expression of CYP3A4 and CYP2C9, and the inhibitory effect on CYP2C9 was much greater than that on CYP3A4, and at the same time, we could also observe that PXD had an inducing effect on the protein expression of CYP3A4 and CYP2C9, therefore, it could be concluded in this work that the PXD could be used as an inducer of CYP3A4 as well as CYP2C9 in rats as well.

As previously mentioned, PXD was metabolized via the CYP3A4 pathway. There are three types of drug inhibition of CYP450 enzymes: competitive inhibition, suicide inhibition, and non-selective inhibition. The intensity of inhibition varies depending on the starting concentration of the inhibitor, the efficiency of the enzyme in metabolizing the inhibitor, and the stability of the inhibitor-enzyme complex. In the present study the inhibition of PXD by CG and silybinin may be nonselective inhibition, which inhibits several CYP isozymes, lacks specificity, and acts when the drug binds nonselectively to the ligand of the ferrous heme portion of the CYP-450 enzyme causing an impairment of the enzyme activity. However, silybinin and CG suppressed PXD metabolism by inhibiting protein expression of CYP3A4 as well as CYP2C9. This also demonstrated that PXD could also be metabolized via the CYP2C9 pathway. All of the above needs to be verified in the context of clinical practice as well as further experimental studies.

## 5. Conclusions

In summary, it was demonstrated that the assay was facile, sensitive, and precise based on the HPLC-UV technique. This bioanalytical approach was employed in the investigation of DDIs in silybinin and PXD as well as CG and PXD rats, and the protein expression in CYP3A4, as well as CYP2C9, was examined in rats using the immunoblotting assay. Silybinin, as well as CG, increased PXD exposure in rats, both of which had a pronounced suppressive effect on PXD metabolism, associated with suppression of protein expression in CYP3A4 as well as CYP2C9. Therefore, the combination of PXD with silybinin and CG should be monitored to avoid clinical adverse effects.

## Data Availability

The original contributions presented in the study are included in the article; further inquiries can be directed to the corresponding author.
